# Supplementary material for: Splice-Junction-Based Mapping of Alternative Isoforms in the Human Proteome
Source: Cell Rep. Author manuscript; Available in PMC 2020 Jan 15. (PMC6961840; doi:10.1016/j.celrep.2019.11.026)
Supplement: 2 [file NIHMS1546469-supplement-2.zip › DF1/PXD010154/Ovary/Ovary_17_HMGB1_ENILACPLVMLR.pdf]

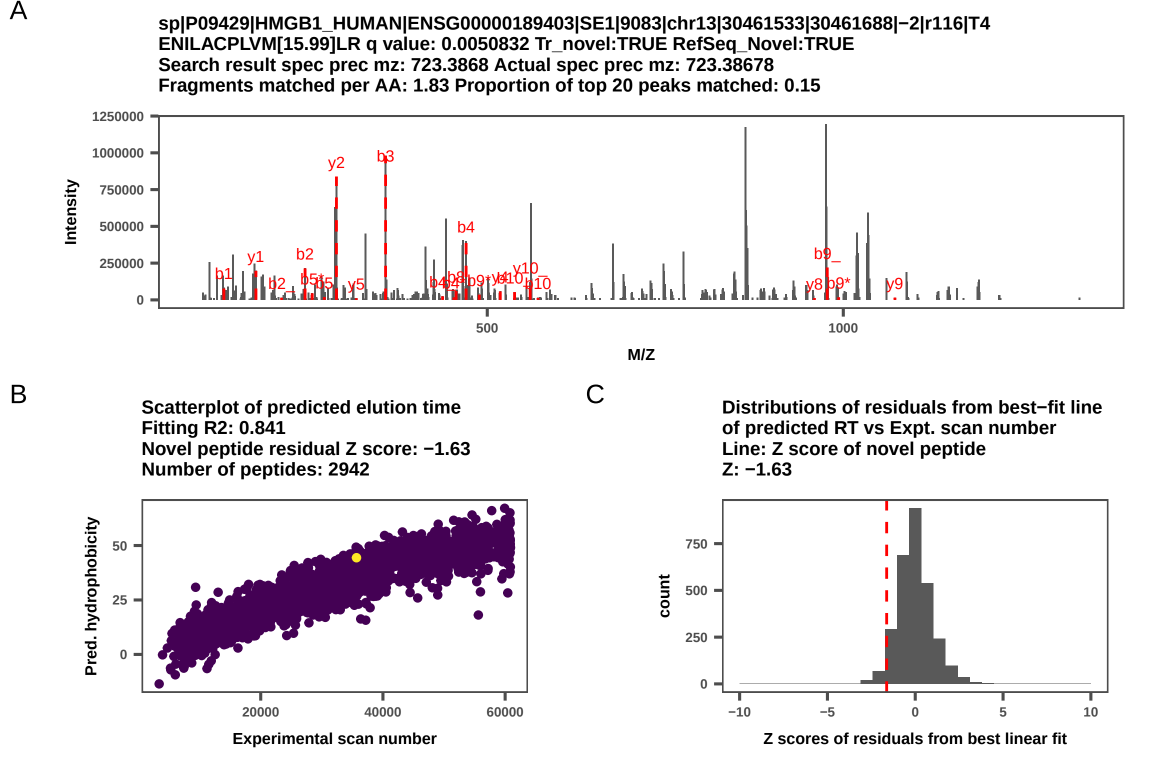
A
sp|P09429|HMGB1_HUMAN|ENSG00000189403|SE1|9083|chr13|30461533|30461688|−2|r116|T4
ENILACPLVM[15.99]LR q value: 0.0050832 Tr_novel:TRUE RefSeq_Novel:TRUE
Search result spec prec mz: 723.3868 Actual spec prec mz: 723.38678
Fragments matched per AA: 1.83 Proportion of top 20 peaks matched: 0.15
Intensity
1250000
1000000
750000
500000
250000
0
M/Z
500
1000
y2
b3
b4
y1
b1
b2
b5*
b5
y5
y10
b10
b9_
y8
b9*
y9
B
Scatterplot of predicted elution time
Fitting R2: 0.841
Novel peptide residual Z score: −1.63
Number of peptides: 2942
Pred. hydrophobicity
50
25
0
20000
40000
60000
Experimental scan number
C
Distributions of residuals from best−fit line
of predicted RT vs Expt. scan number
Line: Z score of novel peptide
Z: −1.63
count
750
500
250
0
−10
−5
0
5
10
Z scores of residuals from best linear fit
